# Integrated case scripts to enhance diagnostic competency

**DOI:** 10.4317/jced.52237

**Published:** 2015-07-01

**Authors:** K. Anbarasi, Phagalvarthy Vijayaraghavan, Sankarapandiyan Sathasivasubramanian, Deivanayagam Kandaswamy

**Affiliations:** 1 Assoc. Prof, Department of Oral Medicine and Radiology, Faculty of Dental Sciences, Sri Ramachandra University, Chennai, India; 2 M.ch. Director, Academics and Administration, Sri Ramachandra University, Chennai, India; 3 M.D.S., Head of the Department, Department of Oral Medicine and Radiology, Faculty of Dental Sciences, Sri Ramachandra University, Chennai, India; 4 M.D.S., Dean, Faculty of Dental Sciences, Sri Ramachandra University, Chennai, India

## Abstract

**Background:**

The overwhelmingly high burden of disease and disorder especially in developing countries requires oral physicians to provide optimal dental treatment without complicating individuals’ general health. The opportunity for learners to extract the multiple aspects of a systemic condition and to relate them with the presenting complaint in order to devise an appropriate dental treatment plan is limited by time in chair- side teaching. To overcome the necessity of exposing students to real patients with varying degrees of underlying disease, those in medical and nursing education unanimously employ imaginary scenarios similar to real cases. However, such clinical scripts are seldom practiced in dental education, and the prospect of structured integration is almost never addressed.

**Objectives:**

To evaluate the effectiveness of applying systematic and integrated case-based discussion in dental education in terms of enhancing five essential skills to novice Indian dental students.

**Methods:**

A mixed- methods study was carried out with thirty graduating third-year students in 5 focus groups. The integrated case-based focused group training occurred in 6 weeks and lasted approximately 90 minutes per discussion. Ten case scripts of hypothetical situations were discussed and five integrated modules were organized as a part of this program. Revised Bloom’s taxonomy was adopted to achieve the expected level of competency.

**Results:**

Students performance following integrated case-based discussions was improved and their acceptance to this practice is positive.

**Conclusions:**

The present study supports the need for course specific, basic science integrated seminars with concurrent case scripts discussion to enhance students’ competencies.

** Key words:**Case scripts, Revised Bloom’s taxonomy, chair-side teaching, integrated teaching.

## Introduction

Connecting general health with oral health and educating dental students to provide appropriate diagnoses and treatment plans for oral diseases are essential part of novice training. To elicit systemic health, sufficient knowledge on basic medical sciences and reasoning skills are essential as trainees assume the role of treatment providers for their patients (1). In most Asian countries, including India, III year bachelor of dental surgery degree (B.D.S.) students are introduced concurrently to general and oral medicine, putting more pressure on the application of medical knowledge for dental patients with systemic illness. Hence, training a novice dentist requires a strong commitment.

At the entry level of clinical practice, learners are only exposed to patients presenting with simple medical histories (2) and they are allowed to treat dental patients with medical complications only during compulsory Rotating Internship (CRI) phase. However, this minimal amount of training is insufficient to prepare students to handle the increasing burden of disease in dental patients effectively and independently. Dewey’s adult learning theory (3,4) makes it clear that learning cannot be disconnected from the reality in which it will be practiced, and the ideal time to learn something is when there is chance to immediately apply the new knowledge. To apply this concept in novice training, we selected the method of using integrated case scripts with prototypical presentations instead of our routine chair-side training in which discussions are focused mainly on oral diseases with little integration of basic medical sciences and the influence of underlying systemic illness on the presenting oral disease. This method encouraged our learners to assemble the data carefully and to develop individualized treatment plans rather than examining the data based on rote memorization.

## Study Desing

-Need assessment.

At the end of academic the year (May, 2014), an in-depth analysis of summative performance of third-year dental students in diagnosis and treatment plan at department level helped us to identify five key aspects are frequently overlooked by learners. Immediate need for the best possible alternate instructional method to enhance learners’ competency was realized ( Table 1, Table 1 (Cont)). We proposed integrated case based training program, discussed with the Director of Academics and Administration of our university, and his guidance was obtained for instrumentation. Ethical approval to design competency based clinical dental education was obtained from the Institutional Review Board of Sri Ramachandra University.

**Table 1:**
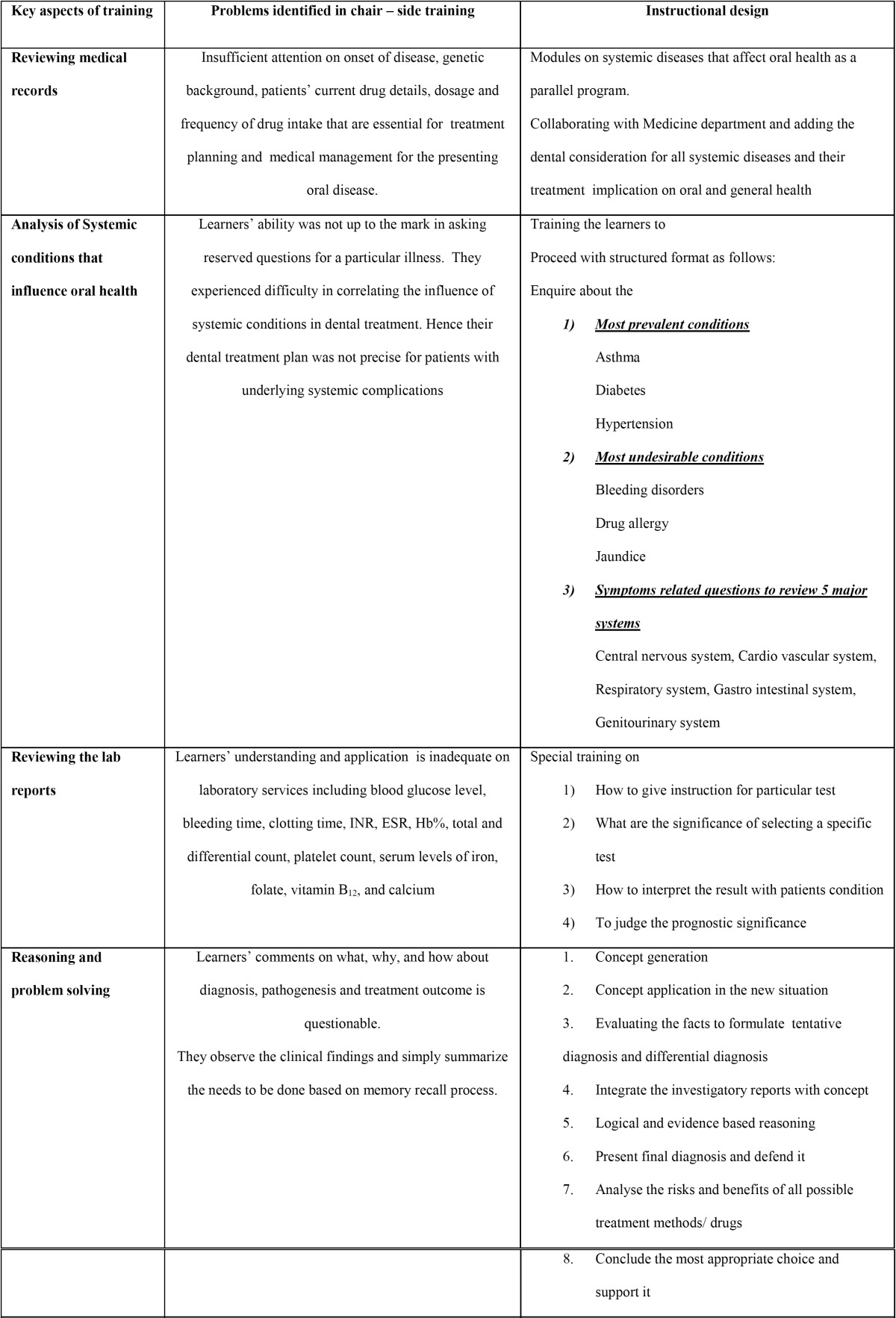
Problems identified and instructional method designed to overcome.

**Table 1 (Cont):**
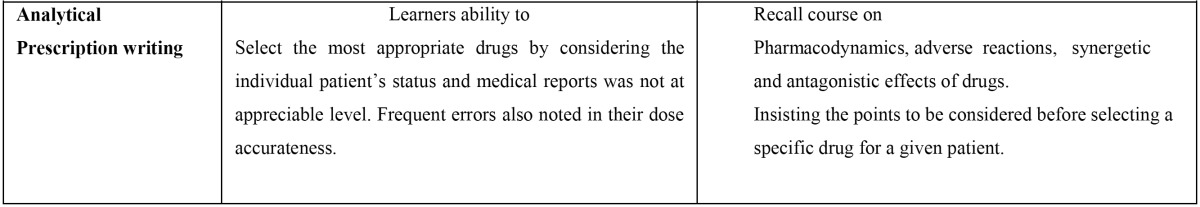
Problems identified and instructional method designed to overcome.

-Program objectives.

The primary focus of our program is to train our undergraduate students to develop necessary diagnostic and treatment planning skills to succeed as independent clinician.

-Process.

The key aspects were shared with our learners through feedback grid and informal conversation. We briefed about our new plan intended to enhance their clinical competency and learners’ willingness to participate in the training program was enquired.

-Method.

A mixed- methods study using focus groups of entry level final year undergraduate dental students was conducted by department of oral medicine and Radiology, faculty of dental sciences, Sri Ramachandra University, in June 2014. We designed five focus groups, each comprising six students selected by alphabetical order to minimize bias. The gender composition included a greater proportion of female students (77% female students versus 23% male students) but all were in same ethnic group and in narrow range of age distribution (21-23 years). The integrated case-based focused group training occurred in 6 weeks and lasted approximately 90 minutes per discussion. Ten case scripts of hypothetical situations with increasing complexity that were drawn from real dental professional contexts was structured by the faculty in charge, reviewed and edited by two peer teachers. The scripts focused on the pragmatic conditions in the interest of helping learners to put their theoretical knowledge into practice by adopting the revised Bloom’s taxonomy (5). Each script was circulated to the students five days before the discussion date. The responsibilities of the course facilitator and the learners were defined as shown in  Table 2. Small group, student centered discussion method was chosen to implement the program, where faculty in-charge acted as a facilitator and monitored students participation. Instructor used to narrate a script with specific dental problem ( Table 3, Table 3 (Cont)) which was composed of many sub problems and initiate the discussion with interchanging ideas. Learners were asked to start their individual preparation once they receive their script and develop a working model by categorizing relevant details for each element of the script. During the discussion phase, learners were asked to share their individual preparation, correlate the positive components of medical history and its significance in the given situation, reason for probing a particular systemic condition, how to proceed the situation, and treatment plan. Subsequently, facilitator will reflect on the key perspectives and stimulate student interactions as well as group decision for the best appropriate solution. In preparing case scenarios, we concentrated on replicating a wide range of dental and oral diseases and utmost care was taken in crafting our experiences to capture students learning on the following five essential skills.

**Table 2:**
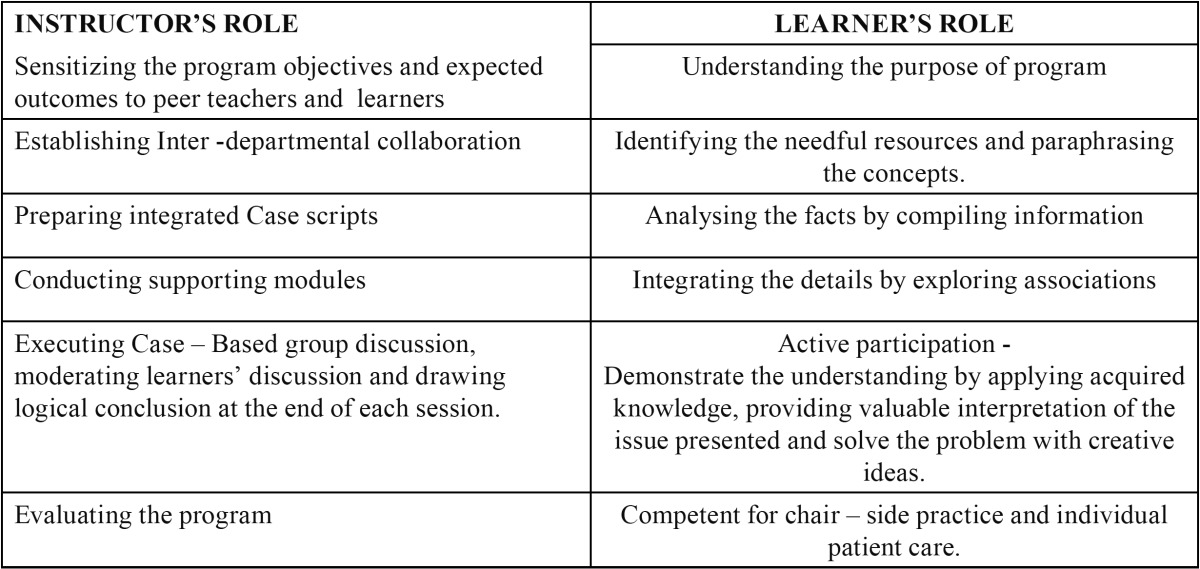
Professional responsibilities of program Instructor and Learner.

**Table 3:**
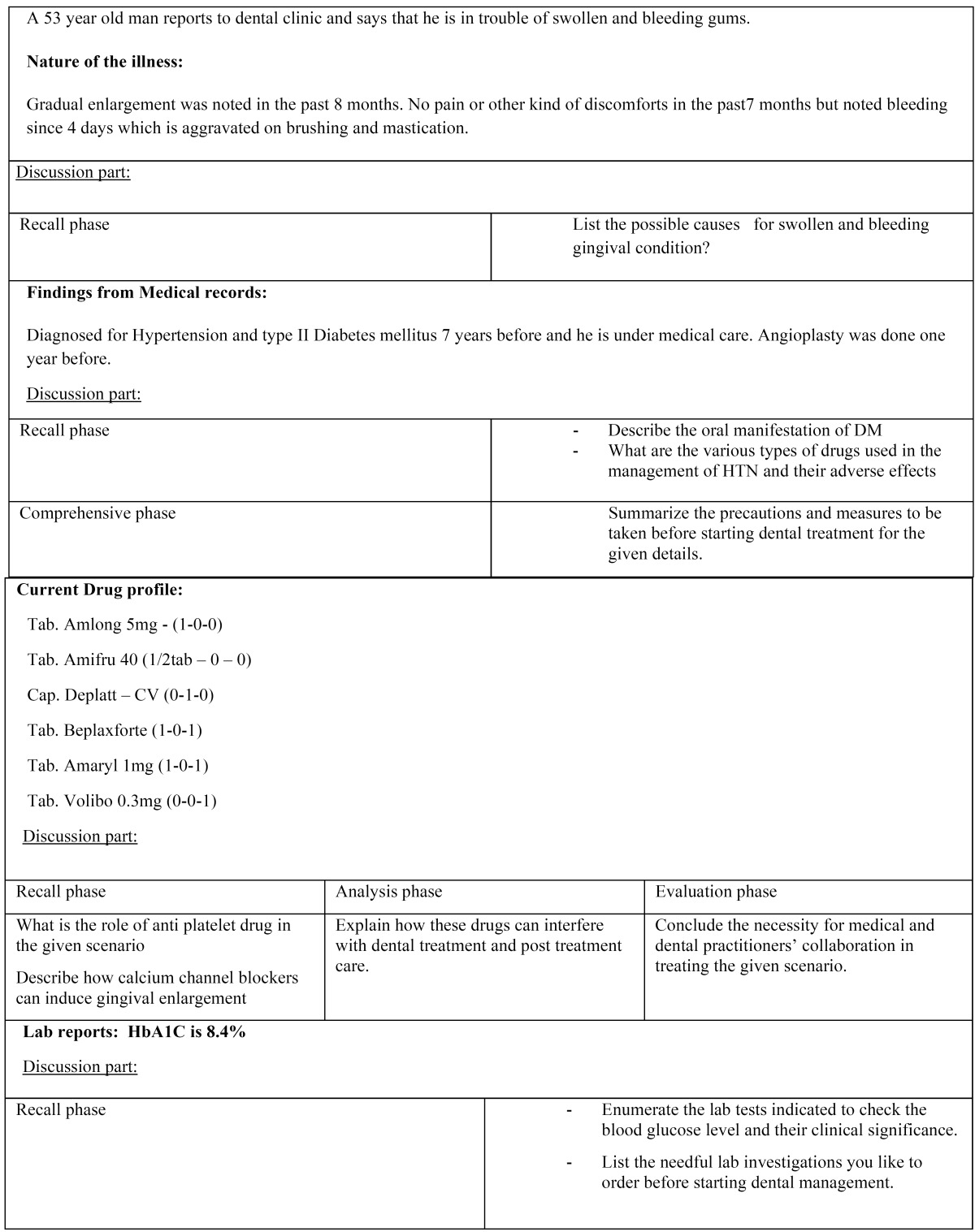
Case script exemplar.

**Table 3(Cont):**
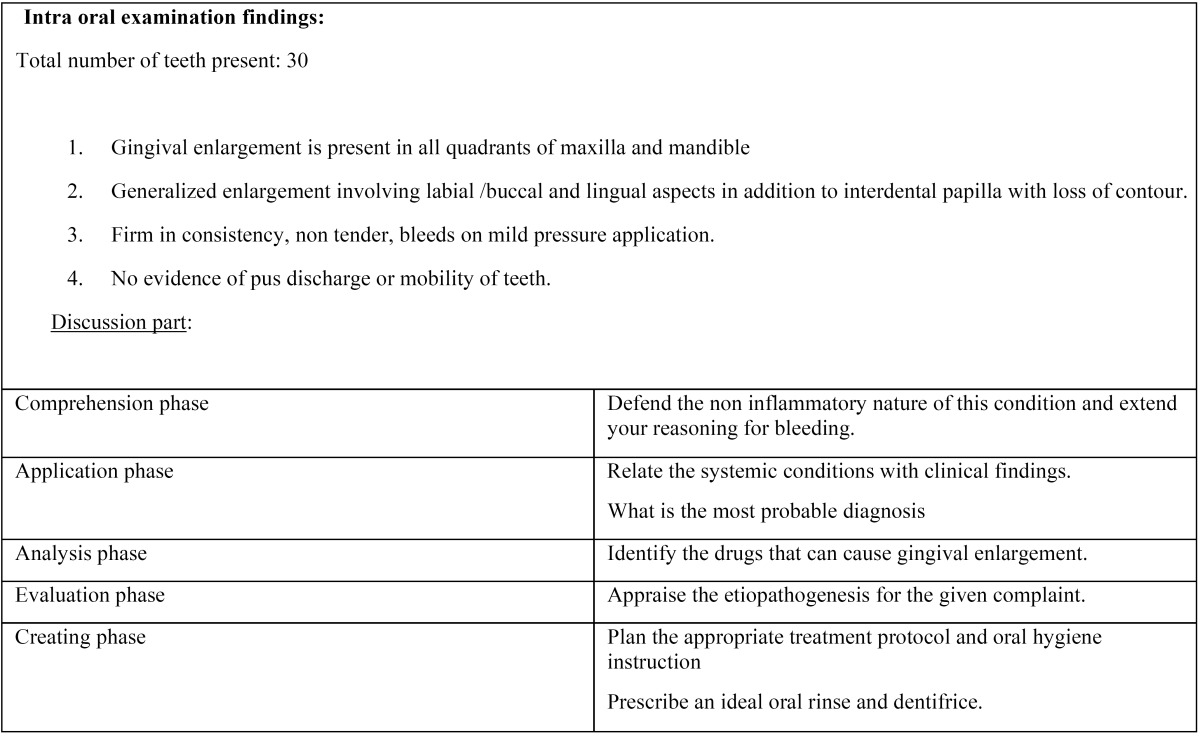
Case script exemplar.

1. Reviewing medical records 

2. Analyzing systemic conditions that influence oral health

3. Lab investigations 

4. Reasoning and problem solving skills

5. Analytical prescription writing 

Five Integrated seminars were conducted to inculcate these skills by integrating basic medical sciences with dental practice.

-Reviewing medical records.

Medical records are an authoritative and cumulative document on past and present illnesses and treatment written by health care professionals caring for the patient (6). It allows the dental professionals to track patients’ medical history and identify problems to provide apposite care. We assisted our learners in gathering the following information from the medical records for efficient dental care.

1. Family history for any genetic disorders

2. Serious illnesses, surgeries or accidents 

3. Risk factors; allergies and drug reactions; current medications and dosage 

-Analysis of systemic conditions that influence oral health.

Essential aspects under this perspective include.

1. Listing the system - specific questions for exploring the issue (mainly for cardiovascular, respiratory, gastrointestinal, genitourinary, haematological and nervous systems) 

2. Focusing on conditions with considerable morbidity and mortality (7).

3. Interpreting how the systemic diseases can interfere with responses to dental treatment and post treatment healing

4. Interpreting the impact of oral disease and its management on systemic condition (8).

-Lab investigations.

The learning outcomes targeted under this objective include

1. Overcoming failures in ordering laboratory tests and interpreting test results

2. Selection of lab tests to enable accurate diagnosis, delivery of appropriate dental treatments, effective monitoring of health status to provide dental care, and the need for physician referral to return the alarming parameters to normal before managing oral diseases

3. Indications and inferences of complete blood count and tests for platelet count, INR, bleeding and clotting time, hepatitis B and C, and HIV to carry out dental procedures in suspected risk groups.

4. Directions to be followed when lab results are “outside the normal range” for a patient seeking dental treatment.

-Reasoning and problem solving skills.

Though it is a cyclic and reflective process that depends on attitude and cognitive application, stepwise training was provided in information collection, addressing relevant information, and logical orientation to derive an action plan in a flexible situation. We encouraged novices to reflect on the information they were receiving and to remain focused on the task.

-Analytical prescription writing.

We took unique interest in the following aspects.

1. Preparation of an essential medicine list (including antibiotics; analgesics; antihistamines; anti-platelets; anticoagulants; corticosteroids; carbamazepine; antacids; antioxidants; vitamin and mineral supplements; and antihypertensive drugs, anti-viral, anti-fungal and anti- inflammatory drugs) 

2. Prescription exercises (9) to build up a hospital wide legibility yardstick.

3. Pharmacodynamics and kinetics of essential drugs, sources of drug information, and critical appraisal of prescribing drugs (10).

## Results

Although our primary research aimed to enhance students’ competency on clinical diagnosis and treatment plan, participants request made us to execute chair side assessment. Following the training program, the participants were taken to clinical side and assessed by detailed case history writing with diagnosis and treatment plan for a given real patient similar to the summative exam. A comparative analysis was made, in which participants’ summative performance on clinical examination was taken as pre-test result and assessment through clinical examination following the new instructional method was taken as post-test result.

1. The overall post test performance was largely on good and excellent level whereas pre test performance was falling in average, and borderline plane.

2. In post test performance, a striking improvement was noted in analysis of systemic condition, reasoning and problem solving skills, whereas significant progress was seen on medical record review and systematic prescription writing. Marginally better performance was observed in interpretation and usage of lab reports.

Learners’ feedback about the program added credibility for two essential features.

Flexibility.

Students unanimously valued the flexibility of the program for developing diagnostic and management skills in a programmed way.

“I liked the integrated approach in a totally new context and in a new system”.

“I am clear about what do I need to know in a composed environment”.

Freedom of fear.

Many students mentioned that the in-house discussions helped them to cross over fear and apprehension in handling patients with systemic illness.

“I determined how I want to proceed when I am handling a patient and how confident and focused I should be”.

## Discussion

Paul R. Lawrence, a renowned professor of Harvard business school described case studies as “a vehicle by which a chunk of reality is brought into the classroom, to be worked over by the class and the instructor”. It is an effective tool to facilitate and assess novice learning in dental education as opposed to direct chair- side learning, which depends heavily on patients’ cooperation and attitude. Classroom ambience allows students to feel secure, going through the trial and error needed for realistic learning. Teacher prepared case scripts are mental representation of portrayal of sequences associated with onset and progress, signs and symptoms, precise or probably related factors of a disease (11). These script based discussions helped our novice students to become competent in diagnosis and treatment planning in an informal, secure, and flexible learning environment.

Many times our students are unable to apply their preclinical knowledge in diagnosis and treatment planning. To emphasize further, a number of dental graduates are uncertain in prescribing drugs on their own. This is because preclinical pharmacology concentrates on system-based pharmacotherapy, with nearly omitting any emphasis on therapeutic knowledge and the skills required for dental practice. In clinical situations, very little time is spent on the principles of drug treatment for a specific situation. This demanded to instruct prescription writing with a revision of clinical pharmacology to avoid medication errors which might be a failure in the treatment process (12). As we conducted inter disciplinary modules as a part of this program, pharmacology, general medicine and dental medicine faculty members presented a practice oriented factual information with hands-on practice in prescription writing, sources of drug information, pharmacovigilance, and essential medicines concept. This resulted in a refined outcome in prescription writing skill during the post test performance.

Researches in medicine and nursing education suggest a link between critical thinking aptitude and accuracy of clinical judgement (13), but nearly no reference about the cognitive components of clinical decision making in dental education literature. Critical thinking is a key factor in initiating the thought processes (14) and move towards a stage of safe dental practice. Our program encouraged students to clarify misconception about short and long term effects of structured and ill-structured health related, and treatment related issues. Students were able to independently identify the oral problems and explore the causative factors and skilfully implement the treatment plan that can likely resolve the problem in their post training practice.

The scope of our program also aims at the pursuit of natural lifelong learning by infusing Bloom’s revised taxonomy (5) in the discussion phase. In practice, theoretical guidance and exposure to real cases occur at different times, and this separates learning from the context in which it is used. In integrated case-based discussion, we were able to support learners in remembering and comprehending the facts learned about pathogenesis, biochemical analysis, pharmacotherapy, and the medical background of systemic diseases and applying them to solve the problem in the given case. This was followed by active analysis of every component of the gathered information, open expression of their ideas, and compiling the information to plan management protocol. The process was contrary to chair- side teaching in which the primary focus is on patient management, and learning assessment is often missed (15).

As an initial step, we concentrated more on content designing and execution levels. To sustain the quality of this instructional method, we plan to develop more structured assessment tools to measure students’ performance and overcome the every possible lacuna.

To sum up:

1. As many dental patients are potentially related to a medical condition (16), history taking should be structured and tutoring to review the medical records should be executed with great attention.

2. Oral diseases with overlapping features and associated systemic pathologies need more analytical courses of action with repeated concept generation, elimination, and fine-tuning to explicate the treatment protocol (17).

3. Laboratory medicine teaching on the topics of test selection and result interpretation is highly valuable for dental students as decision supporting tools (18).

4. Pharmacotherapy should be approached practically with more emphasis on dental grounds.
